# Behind the Numbers: Cancer Survivor Recruitment by Demographics and Clinical Status

**DOI:** 10.1002/cam4.71782

**Published:** 2026-04-17

**Authors:** Michelle Y. Martin, Wendy Demark‐Wahnefried, Iman Omairi, Yu‐Mei Schoenberger‐Godwin, Robert A. Oster, Kelly Kenzik, Nataliya V. Ivankova, Dori Pekmezi, Kevin Fontaine, Laura Q. Rogers, Maria Pisu

**Affiliations:** ^1^ Department of Preventive Medicine University of Tennessee Health Science Center Memphis Tennessee USA; ^2^ Department of Nutrition Sciences, School of Health Professions University of Alabama at Birmingham Birmingham Alabama USA; ^3^ O'neal Comprehensive Cancer Center University of Alabama at Birmingham Birmingham Alabama USA; ^4^ Department of Medicine, Heersink School of Medicine University of Alabama at Birmingham Birmingham Alabama USA; ^5^ Department of Surgery Boston University Chobanian and Avedisian School of Medicine Boston Massachusetts USA; ^6^ Department of Health Services Administration, School of Health Professions University of Alabama at Birmingham Birmingham Alabama USA; ^7^ Department of Health Behavior, School of Public Health University of Alabama at Birmingham Birmingham Alabama USA

**Keywords:** cancer, recruitment, survivors

## Abstract

**Background:**

Recruiting cancer survivors to research studies requires multiple steps that may be differentially challenging across groups of survivors.

**Aims:**

We examined associations between progression through recruitment steps and sociodemographic and clinical characteristics of survivors invited to participate in the Adapting MultiPLe behavior Interventions that eFfectively Improve (AMPLIFI) Cancer Survivor Health randomized control trial of web‐based healthy lifestyle interventions.

**Materials and Methods:**

Survivors from two population‐based cancer registries and a hospital registry (*n* = 23,270) were mailed an AMPLIFI invitation letter then called by recruiters. Outcomes included being (1) reached by recruiters, (2) screened for eligibility, (3) deemed eligible, and (4) consented. Bivariate and logistic regression analyses examined associations with sociodemographic and clinical factors.

**Results:**

7742 (33.3%) survivors were reached. Being reached was positively associated with being female, age 65+, African American, urban resident, and < 2 years from cancer diagnosis; negatively associated with colorectal cancer. Among those reached, 7438 were offered screening and 2278 (30.6%) were screened. Being screened was positively associated with being female, African American, and < 2 years from diagnosis; negatively associated with age 65+, resident in high disadvantage areas, survivor of colorectal cancer. Of survivors screened, 716 (32%) were eligible. Being eligible was positively associated with < 2 years from diagnosis; negatively associated with colorectal and kidney cancer. Of those eligible, 438(61.2%) consented to participate. Being consented was negatively associated with being African American and from high disadvantage areas.

**Discussion and Conclusion:**

Outcomes across recruitment steps vary by survivors' characteristics indicating that opportunities exist for tailored approaches to promote greater overall diversity in clinical trials.

## 1 Introduction

Successfully recruiting cancer survivors to clinical trials is critical to achieve the statistical power needed to evaluate study hypotheses; however, a leading reason for trial failure is low accrual [1]. While research participation is low for survivors overall, the variation in participation rates across clinical and sociodemographic groups suggests an even greater concern [2, 3, 4, 5, 6, 7]. Survivors who are older, from minority and/or from lower socioeconomic status groups, and from rural areas, are historically underrepresented in cancer research. Recruiting survivors of certain cancer types (e.g., colorectal [CRC]) also is met with mixed success [8]. A lack of diversity in clinical trial participation limits the generalizability of study findings and undermines public health efforts to improve health outcomes for all.

Population‐based recruitment through cancer registries is a well‐accepted strategy for engaging cancer survivors in research [9]; however, prior studies suggest it has differential success in recruiting diverse survivors [10, 11, 12, 13]. Pinto and colleagues, for example, found that recruiting through tumor registries resulted in more highly‐educated and higher income participants compared to other types of targeted mailings (e.g., hospital/clinic mailings) [14]. The population‐based recruitment process requires the completion of many steps, including reaching participants often by mail and telephone, screening for eligibility once reached, and consenting them if, and when, eligibility is established. To achieve clinical and sociodemographic diversity of the cancer survivor population in research, it is crucial to identify where along the recruitment steps we “lose” survivors and if this attrition varies by survivor characteristics. However, many studies do not report this level of detail and focus instead on the overall recruitment yield, limiting the ability to develop appropriate strategies at multiple, sequential steps to achieve that goal.

To address this gap in knowledge we used data from recruitment into a large program project that integrated three randomized controlled trials into one for efficiency and scientific yield (Adapting MultiPLe behavior Interventions that eFfectively Improve (AMPLIFI) Cancer Survivor Health, P01 CA29997; ClinicalTrials.gov, NCT04000880) [15]. The integrated trial tested three AiM Plan and act on LIFstYles (AMPLIFY) web‐based intervention modules which promoted diet only, exercise only, or diet plus exercise combined, in older cancer survivors who completed primary treatment [15]. The aim of this study was to examine whether demographic and clinical characteristics were associated with progressing through each step of the recruitment process.

## 2 Methods

AMPLIFI was funded by the National Cancer Institute. This program project was a collaborative effort between the University of Alabama at Birmingham and the University of Tennessee Health Science Center (P01 CA29997). The UAB Institutional Review Board is the single IRB for this study (IRB‐300002068). Participants were recruited from the UAB hospital registry and several state registries (Alabama, North Carolina, Texas, Michigan, and Tennessee). For this analysis, we focused on the UAB hospital registry and two of the state registries (Alabama Statewide Cancer Registry, North Carolina State Registry) as their state approved approaches for recruiting survivors were similar. After appropriate required approvals, the registries provided names and contact information for survivors who met AMPLIFI eligibility criteria for age (≥ 50 years old) and cancer type (diagnosis of early‐stage multiple myeloma or non‐Hodgkin lymphoma, localized renal or ovarian cancer, or locoregional cancers of the colorectum, prostate, endometrium, thyroid, or female breast) to the research team who was responsible for contacting, screening, and consenting them. The first step of the process was to send an AMPLIFI invitation letter to survivors describing the research opportunity and informing them that a recruiter would call to describe the study and assess their interest in participation. Survivors could opt out of this call by leaving a message on a toll‐free number. Recruiters called survivors who did not opt out, attempting to reach them up to seven times at varying times and days. Individuals successfully reached could learn more about the study and, if interested, were screened to determine eligibility.

Screening assessed additional eligibility criteria: completion of primary cancer treatment, no evidence of cancer recurrence or progressive cancer, body mass index (BMI) = 25–50 kg/m^2^, < 150 min/week of aerobic physical activity, at least 8th grade education, English‐speaking and writing, and residing in an area that receives wireless coverage. Survivors who were already participating in another exercise, diet, or weight loss program, lived in a skilled or assisted living facility, or had conditions that precluded participation in AMPLIFY interventions were excluded. The complete inclusion and exclusion criteria have been published previously [15]. If eligible, survivors were invited to enroll and provide consent.

### 2.1 Study Outcomes

We included four binary outcomes: (1) Reached, individuals who were successfully contacted by telephone and spoke to recruiters [not reached included survivors for whom there was no answer after multiple attempts, those with disconnected or incorrect phone numbers, or those who were deceased (we learned of some survivors' deaths at the time of the recruitment call)]; (2) Screened, individuals who were reached and completed eligibility screening. Survivors were not offered screening and were excluded from the denominator if they indicated that they did not have cancer (*n* = 106), did not speak English (*n* = 23), or were mentally/physically incapable of completing the screening (*n* = 176); (3) Eligible, survivors who screened eligible for the AMPLIFI trial; and (4) Consented, survivors who were eligible for and provided consent to participate.

### 2.2 Sociodemographic and Clinical Factors

From cancer registry records we abstracted sex (female/male), age (< 65 or ≥ 65 years old), race (White/African American), time since diagnosis (< 2 years since diagnosis, 2–5 years after diagnosis, and 5+ years since diagnosis), and cancer type (breast, prostate, CRC, endometrium, kidney), and “other” cancers (ovarian, multiple myeloma, non‐Hodgkin lymphoma, and thyroid cancers). Physical addresses were geocoded to obtain census tracts and blocks, and linked to: (i) the US Department of Agriculture's Rural Urban Commuting Area (RUCA) codes to define residence in urban (RUCA code 1) versus nonurban areas (RUCA codes > 1), and (ii) the Area Deprivation Index (ADI) to define residence from lowest (ADI 0%–20%) to highest (ADI > 80%–100%) socioeconomic disadvantage. Additional sociodemographic information was obtained during screening, that is, education (high school or less, some college or more) and family's annual income (< 25 K, 25 to < 50 K, 50 to < 75 K, 75 K or more).

### 2.3 Statistical Analyses

Descriptive statistics were used to characterize the sample. Chi‐Squared tests (or Fisher's exact test for cells < 5) were used to assess if the observed frequencies within the sociodemographic and clinical variables differed. Logistic regression analyses were adjusted for all covariates (sex, age, race, urban/nonurban status, cancer type, time since diagnosis, and ADI, and additionally education and income for outcomes eligible and consented) and used to generate odds ratios and 95% confidence intervals. Some of the covariates were dichotomized based on findings from descriptive analyses, that is, cancer type (CRC/kidney vs. non‐CRC/kidney cancers), (≥ some college vs. lower education), income (≥ $75,000 vs. lower income), and socioeconomic disadvantage (highest ADI [> 80%–100%] vs. lower). Statistical tests were two‐sided, and declared statistically significance at *p* ≤ 0.05. SAS 9.4 (Cary, NC) was used for analysis.

## 3 Results

Recruitment letters were sent to 23,270 survivors who were mostly female (58%), ≥ 65 years old (61%), White (~70%), and within 2–5 years post diagnosis (~58%). About half (53%) were urban dwellers and almost 39% were survivors of breast cancer (Table 1). Fifteen survivors actively opted out.

**TABLE 1:** Sociodemographic and clinical characteristics of survivors mailed an invitation letter for participation in AMPLIFI trial (*N* = 23,270).

Characteristic	*N* (%)
**Gender**	
Female	13,551 (58.2)
Male	9719 (41.8)
**Age categories**	
< 65	9064 (39.0)
≥ 65	14,206 (61.0)
**Race/ethnicity**	
Non‐Hispanic White	16,267 (69.9)
African American	7002 (30.1)
**Rural/urban**	
Urban	11,583 (53.2)
Nonurban	10,203 (46.8)
Missing	1484 (6.4)
**Time since diagnosis**	
< 2 years	3971 (17.1)
2–5 years	13,451 (58.1)
5+ years	5749 (24.8)
Missing	99 (0.4)
**Cancer type**	
Breast	8979 (38.6)
Prostate	6473 (27.8)
Colorectal	3283 (14.1)
Endometrium	1998 (8.6)
Kidney	1428 (6.1)
Other cancers^a^	1109 (4.8)
**Area Deprivation Index quintiles**	
ADI1 (0–20)	1153 (4.9)
ADI2 (21–40)	2751 (11.8)
ADI3 (41–60)	4297 (18.5)
ADI4 (61–80)	6059 (26.0)
ADI5 (81–100)	7320 (31.5)
Missing	1690 (7.3)

^a^ Ovarian cancer, thyroid, non‐Hodgkin lymphoma and multiple myeloma.

**FIGURE 1:**
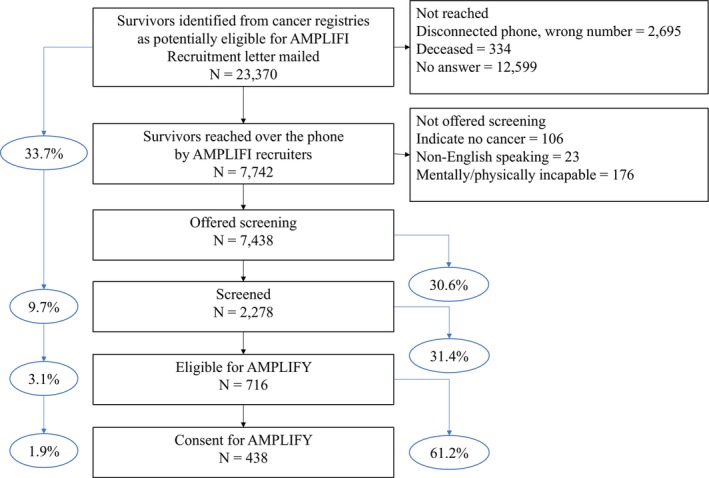
Flow chart of the recruitment process.

**FIGURE 2:**
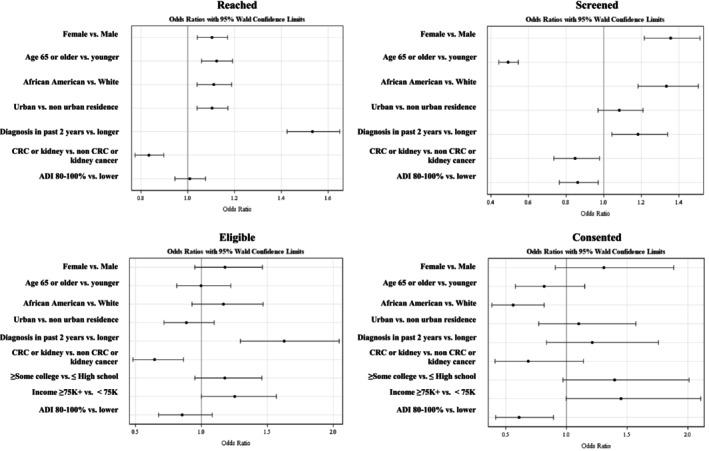
Results of adjusted logistic regression analyses, odds ratios (or) and 95% confidence intervals (CI).

Among survivors who were sent a letter, 33% (7742) were successfully reached by telephone (Figure 1, Table 2). Of the 15,528 not reached, 2695 (17.4%) had disconnected phones or wrong phone numbers, 334 (2.15%) were deceased, and for the rest, the calls were not answered (Figure 1). Survivors who were female, older (≥ 65), African American, urban, and closer to diagnosis were more likely to be reached than their counterparts (*p*‐values < 0.05) (Table 2). Survivors with cancers categorized as “other” (ovarian cancer, thyroid, lymphoma, and multiple myeloma) were more likely to be reached (40.5%) and CRC survivors were the least likely to be reached (28.4%) (*p* < 0.001). Across ADI quintiles, the proportion reached ranged from 32.5% to 33.9% (*p* = 0.002). Significant associations were largely confirmed in adjusted logistic regression analyses (Figure 2).

**TABLE 2:** Characteristics of cancer survivors reached by phone by recruiter among 23,370 approached by letter, and of survivors screened to verify eligibility among 7438 offered screening.

Characteristic	Reached *N* (%^a^)	*p* ^b^	Screened *N* (%^a^)	*p* ^b^
**All**	**7742 (33.7)**	**—**	**2278 (30.6)**	**—**
**Gender**				
Female	4626 (34.1)	0.001	1485 (33.3)	< 0.001
Male	3116 (32.1)		793 (26.6)	
**Age categories**				
< 65	2931 (32.3)	0.016	1158 (40.7)	< 0.001
≥ 65	4811 (33.9)		1120 (24.4)	
**Race/ethnicity**				
Non‐Hispanic White	5258 (32.3)	< 0.001	1438 (28.3)	< 0.001
African American	2484 (35.5)		840 (35.6)	
**Rural/urban**				
Urban	4039 (34.9)	< 0.001	1268 (32.7)	< 0.001
Nonurban	3249 (31.8)		895 (28.6)	
Missing	454 (30.6)		115 (26.6)	
**Time since diagnosis**				
< 2 years	1644 (41.4)	< 0.001	550 (34.7)	< 0.001
2–5 years	4453 (33.1)		1228 (28.8)	
5+ years	1601 (27.8)		482 (31.3)	
Missing	44 (44.4)		18 (0.8)	
**Cancer type**				
Breast	2984 (33.2)	< 0.001	996 (34.2)	< 0.001
Prostate	2224 (34.4)		581 (27.2)	
Colorectal	931 (28.4)		209 (23.9)	
Endometrium	713 (35.7)		202 (29.8)	
Kidney	441 (30.9)		133 (32.0)	
Other cancers^c^	449 (40.5)		157 (37.3)	
**Area Deprivation Index**				
0%–20%	963 (33.9)	0.002	145 (33.5)	0.01
> 20%–40%	890 (32.5)		306 (35.5)	
> 40%–60%	1423 (33.1)		431 (31.4)	
> 60%–80%	1992 (32.9)		566 (29.4)	
> 80%–100%	2474 (33.8)		702 (29.8)	
Missing	517 (30.6)		128 (25.9)	

^a^ Values are row percentages (% Distribution by category).

^b^ Chi Squared or Fisher Exact test.

^c^ Other cancers = ovarian, multiple myeloma, non‐Hodgkin lymphoma, and thyroid cancer.

Among survivors reached, 7438 were offered eligibility screening and 30.6% (2278) were screened (Figure 1, Table 2). Survivors more likely to be screened were female, African American, urban residents, and closer to diagnosis (*p* ≤ 0.001) (Table 2). Older survivors (≥ 65) were less likely to be screened than younger survivors (*p* < 0.001). Breast cancer survivors (34.2%) or survivors of “other” cancers (37.3%) were most likely to be screened, while CRC survivors were least likely (23.9%) (*p* < 0.001). From the lowest to the highest ADI quintile, 33.5%–29.8% of survivors agreed to be screened (*p* = 0.01). Significant associations were confirmed in adjusted analyses, except for urban versus nonurban residence (OR 1.08, CI 0.97–1.21) (Figure 2).

Of survivors screened, 32% (716) were eligible for AMPLIFI (Figure 1, Table 3), with no significant differences by sex, age, race, urban versus nonurban residence, or ADI. Survivors closer to diagnosis and survivors of breast cancer were more likely to be eligible (*p* < 0.001), while survivors of CRC and kidney cancer were less likely (*p* = 0.03). Moreover, survivors with some college or having graduated college, or with higher incomes, were more likely to be eligible than their counterparts (*p* < 0.05). In adjusted analyses, significant associations were confirmed for income but not for education (OR 1.18, CI 0.95–1.46) (Figure 2).

**TABLE 3:** Characteristics of eligible cancer survivors among 2278 screened, and who consented to participate among 716 eligible.

Characteristics	*N* (% ^a^) eligible	*p* ^b^	*N* (%^a^) consented	*p* ^b^
**All**	**716 (31.4)**	**—**	**438 (61.2)**	
**Gender**				
Female	486 (32.7)	0.068	311 (64.0)	0.024
Male	230 (29.0)		127 (55.2)	
**Age categories**				
< 65	385 (33.2)	0.058	249 (64.7)	0.038
≥ 65	331 (29.5)		189 (57.1)	
**Race**				
Non‐Hispanic White	435 (30.2)	0.113	296 (68.0)	< 0.001
African American	281 (33.4)		142 (50.5)	
**Rural/urban**				
Urban	403 (31.8)	0.937	255 (63.3)	0.395
Nonurban	283 (31.6)		170 (60.1)	
Missing	30 (26.1)		13 (43.3)	
**Time since diagnosis**				
< 2 years	209 (38.0)	< 0.001	131 (62.7)	0.372
2–5 years	378 (30.8)		232 (61.4)	
5+ years	118 (24.5)		65 (55.1)	
Missing	11 (61.1)		10 (90.9)	
**Cancer type**				
Breast	337 (33.8)	0.031	218 (64.7)	0.170
Prostate	182 (31.3)		99 (54.4)	
Colorectal	50 (23.9)		27 (54.0)	
Endometrium	66 (32.7)		45 (68.2)	
Kidney	31 (23.3)		19 (61.33)	
Other Cancers^c^	50 (31.8)		30 (60.0)	
**Education**				
Some high school or less	21 (22.3)	0.034	4 (19.0)	< 0.001
High school	150 (36.1)		72 (48.0)	
Some college	235 (37.5)		151 (64.3)	
College graduate	305 (37.4)		209 (68.5)	
Missing	5 (1.5)			
**Income**				
< 25 K	119 (29.0)	0.002	53 (44.50)	< 0.001
25 to < 50 K	168 (38.6)		102 (60.7)	
50 K to < 75 K	119 (38.1)		75 (63.0)	
75 K and more	261 (40.1)		182 (69.7)	
Missing	49 (10.4)		26 (53.0)	
**Area Deprivation Index**				
0%–20%	48 (33.1)	0.200	38 (79.2)	< 0.001
> 20%–40%	94 (30.7)		65 (69.1)	
> 40%–60%	136 (31.5)		91 (66.9)	
> 60%–80%	200 (35.3)		127 (63.5)	
> 80%–100%	204 (29.1)		101 (49.5)	
Missing	34 (26.6)		16 (47.1)	

^a^ Values are row percentages (% Distribution by category).

^b^ Chi Squared or Fisher Exact test.

^c^ Other cancers = ovarian, multiple myeloma, non‐Hodgkin lymphoma, and thyroid cancer.

Among eligible survivors, consent was provided by 61.2% (438) (Figure 1, Table 3). Survivors more likely to consent were female, < 65 years old, with higher education and income (*p* < 0.05). African American survivors (*p* < 0.001) and those from high ADI areas were less likely to consent (*p* < 0.001) than their counterparts. No significant differences were found by urban vs. nonurban residence, time since diagnosis, and cancer type. Adjusted analyses confirmed African Americans (OR 0.56, CI 0.39–0.82) and survivors in high ADI areas (OR 0.61, CI 0.42–0.89) were less likely to consent, but other associations by gender, age, education, and income were not statistically significant (Figure 2).

## 4 Discussion

Increasing representation of clinical trial participants is a scientific priority [16]. Despite cancer registries offering the best opportunity for recruiting representative participant samples, recruitment from these sources is challenging because of the multistep process that presents multiple opportunities for attrition: overall, we recruited < 2% of survivors from our initial pool of potential study participants. Our study provides convincing evidence that clinical and sociodemographic factors are associated with each step of the recruitment process, but not always consistently in the same direction. Below we highlight our findings by factors. We conclude our paper by offering practical implications for facilitating the recruitment of participants to research studies.

### 4.1 Clinical Factors

Individuals with CRC/kidney cancer in our study were less likely than all others to be reached, screened, and/or eligible, although not less willing to consent than survivors of other cancers. We observed the same pattern for survivors who were 2 or more years from diagnosis compared to survivors with more recent diagnoses. These findings suggest that clinical characteristics need to be considered when designing recruitment strategies in which a one‐size fits all approach will likely falter across different cancer types and/or times since diagnosis. The data on whether cancer type predicts research participation is mixed. In analysis of three diet and exercise intervention trials [2], cancer type was not associated with willingness to participate in two of the trials, while in the third trial prostate and breast cancer survivors were more willing to participate than CRC survivors, and breast cancer survivors were more willing than prostate cancer survivors. Understanding the reasons for these differences will help refine recruitment approaches to be attractive and feasible for all cancer survivors. Our finding that it was more difficult to reach survivors 2 or more years postdiagnosis may be explained, in part, by outdated contact information. While cancer registries are a great source of potential participants, more than 90% of the survivors with disconnected telephone numbers were 2 or more years postdiagnosis at the time we mailed the AMPLIFI invitation letter. Reaching longer term survivors may require recruitment strategies other than, or in conjunction with, cancer registry approaches.

### 4.2 Sociodemographic Factors

Our success in reaching women more so than male survivors by telephone was unexpected. Marketing research suggests that women are less likely to answer telephone calls from unrecognized numbers [17]. However, our findings may be explained by a study that found men to be less responsive to direct mail recruitment compared to other strategies, for example, referral by primary care provider [18]. Our recruitment approach (beginning with a mailed letter, followed by a telephone call) may have resonated less with males. Future research should investigate whether a higher level of physician involvement is needed to increase reach and willingness of men to be screened for research studies. Because men are just as likely to enroll as women once screened and found to be eligible [18], we must solve the challenge of how to increase the willingness of men to consider and screen for research participation.

Age also played a role in our recruitment with older survivors being more likely to be reached, but less willing to be screened and less likely to consent to participate compared to younger peers. The pattern of findings remained the same in adjusted analyses with one exception: consent no longer differed by age. Several factors may explain why older adults had less interest in being screened. In our study, a lack of interest in a web‐based intervention that required physical activity may have influenced willingness to be screened. These reasons would be consistent with an analysis of clinical trial screening logs within the National Cancer Institute Community Oncology Research Program, which showed that a lack of desire to participate in studies and a concern for adverse events were the most common reasons older survivors declined research participation [19].

After adjustment for covariates, African American cancer survivors were more likely to be reached, willing to be screened, and just as likely to be eligible for the trial. Despite this engagement across the early recruitment steps, African American survivors were less willing to consent than White survivors. Our finding is in contrast to a meta‐analysis showing that African American cancer survivors participated in cancer clinical trials at the same rate as White survivors [20], but consistent with a study in which Black cancer survivors expressed less willingness and actual research participation [21]. These inconsistent findings reflect the complexity of recruiting this population. In the work by Wenzel and colleagues, African American cancer survivors reported a sense of obligation to participate in research that generates knowledge about reducing disparities, while simultaneously reporting feelings of mistrust in research [22]. This may be reflected in our study by African American survivors completing the earlier steps of our recruitment process, but then not agreeing to consent. Studies to fully understand the factors and the context that influence decision‐making and research participation for this population are still needed.

Recruiters for our study were less successful in reaching survivors from nonurban areas and when reached, these survivors were less willing to be screened. Notably, more than 40% of survivors in nonurban areas were also living in highly disadvantaged areas, compared to only a quarter of survivors living in urban areas. Urban/nonurban differences in screening may be explained by this prevalent disadvantage that may impact how survivors consider and evaluate research participation. This is supported by our finding that higher disadvantage was significantly associated with lower odds of consenting despite equal odds of being eligible. Moreover, in the fully adjusted models that included a measure of area disadvantage, there was no longer any difference in willingness to be screened between rural and nonurban survivors. Therefore, if we are to fully realize geographic diversity in cancer clinical trials, targeted efforts may be needed to reflect the challenges associated with cancer survivors' living contexts.

Our study has limitations. First, the analyses include survivors from only two southeastern state cancer registries and one hospital registry, limiting the generalizability of findings and reducing the racial and ethnic diversity of our sample. Second, recruitment activities occurred during COVID‐19, and it is unclear whether response to our recruitment efforts was influenced by the pandemic. Third, an ancillary trial designed to increase the participation of African American survivors in research (R01CA242737) was appended to AMPLIFI and may have influenced our outcomes. Fourth, survivors with disconnected numbers or decedents were largely identified when we attempted to reach them by telephone. While this provides important information about the percentage of survivors from cancer registries who cannot be contacted, we acknowledge that this reality also leads to selection bias. Finally, multiple testing is a limitation, and as the sample size decreased across outcomes, our ability to detect differences may have been compromised.

Our project also has many strengths. We are among the few to shift from “all‐or‐nothing” thinking about recruitment to viewing recruitment as a process with multiple sequential steps and considering multiple outcomes along this process. Our findings that clinical and sociodemographic factors are associated with “losing” cancer survivors along these steps suggest that increasing research enrollment may require developing and testing stage‐specific recruitment strategies that are also tailored to survivor characteristics. Some of the inconsistent patterns in our study represent the most intriguing findings and serve as a second study strength. Certain demographic groups show opposite patterns of engagement in the recruitment process at different steps, for example, African American or older survivors. These findings advance recruitment science and demonstrate that barriers to recruitment operate differently across recruitment steps. A third study strength is our population‐based approach to recruitment. Using cancer registries with a specific list of cancer survivors provided a denominator for our analyses and allowed us to assess how successful we were in reaching different sociodemographic and clinical groups.

In addition to these strengths, our study has practical implications for each step in the recruitment process. Routine updates to cancer registries to reflect the current vital status and, if feasible, contact information of survivors will help focus recruitment efforts saving resources (e.g., staff time) and avoiding unintended harm (e.g., the distress caused by contacting decedents' loved ones). With our finding that there were differences in who was reached, future recruitment efforts would likely benefit from tailored approaches to reach all survivors. These tailored approaches might include sending different letters of invitation. For example, given literature that suggests that men are more responsive to physician recommendations for research participation, invitation letters sent to men that includes a physician's signature may be more effective in increasing reach. Once a potential participant is successfully reached, finding novel ways to create enough interest in the research opportunity and/or facilitate decision making, will be important to increase the willingness of individuals to be screened for study eligibility. Traditional recruitment calls often begin with presenting the research study, assessing interest in participation, then proceeding with eligibility screening if the participant is willing. An alternative approach is to present the research project followed by a discussion with the potential participant about the pros and cons of participating in the study. This approach can help explore ambivalence in research participation and permits a conversation that honors participant autonomy and motivation. For example, in a study by Scarinci et al., African Americans with no cancer history expressed willingness to participate in nontherapeutic cancer research studies, with top motivators being education, self‐benefit, benefit to others, and curiosity [23]. Identifying motivators for research participation and integrating those reasons in the recruitment calls may increase interest in research opportunities. Eligibility is also a recruitment step that may prove to be a barrier to recruitment. Reasons for exclusion for certain groups of survivors, for example, individuals with low income, should be continuously monitored and eligibility criteria revised to be more inclusive, if indicated, while maintaining study rigor. The consent process could also be improved. Personalized approaches to obtaining consent that consider patient level factors that influence decision‐making may be important to recruiting a diverse study sample [24]. Researchers should routinely report the reasons why participation is declined. While many studies collect this information, it is rarely shared with the broader scientific community, limiting our ability to act on that information to inform how we design and recruit to research studies.

In conclusion, while cancer registries may remain the best population‐based recruitment strategy, this approach does not guarantee that recruited samples will be demographically and clinically diverse. Framing recruitment as a step‐based process and understanding the factors that drive decision making at each step for potential participants is a conceptual advancement that is needed to move the field forward.

## Author Contributions

**Michelle Y. Martin:** conceptualization (equal), formal analysis (equal), investigation (equal), methodology (equal), project administration (equal), supervision (equal), writing – original draft (lead). **Wendy Demark‐Wahnefried:** conceptualization (supporting), funding acquisition (lead), investigation (supporting), project administration (equal), writing – review and editing (equal). **Iman Omairi:** formal analysis (equal), writing – review and editing (equal). **Yu‐Mei Schoenberger‐Godwin:** conceptualization (supporting), investigation (supporting), writing – review and editing (equal). **Robert A. Oster:** conceptualization (supporting), formal analysis (supporting), writing – review and editing (equal). **Kelly Kenzik:** conceptualization (supporting), investigation (supporting), writing – review and editing (equal). **Nataliya V. Ivankova:** conceptualization (supporting), investigation (supporting), writing – review and editing (equal). **Dori Pekmezi:** conceptualization (supporting), investigation (supporting), writing – review and editing (equal). **Kevin Fontaine:** conceptualization (supporting), investigation (supporting), writing – review and editing (equal). **Laura Q. Rogers:** conceptualization (supporting), investigation (supporting), writing – review and editing (equal). **Maria Pisu:** conceptualization (equal), data curation (lead), formal analysis (equal), investigation (equal), methodology (equal), project administration (equal), supervision (lead), writing – review and editing (equal).

## Funding

This work was supported by National Cancer Institute (CA229997, P30 CA013148), American Cancer Society (CRP‐19‐175‐06‐COUN).

## Conflicts of Interest

The authors declare no conflicts of interest.

## Data Availability

The data that support the findings of this study are available from the corresponding author upon reasonable request.
